# Characteristics of Patients Using Different Patient Portal Functions and the Impact on Primary Care Service Utilization and Appointment Adherence: Retrospective Observational Study

**DOI:** 10.2196/14410

**Published:** 2020-02-25

**Authors:** Xiang Zhong, Jaeyoung Park, Muxuan Liang, Fangyun Shi, Pamela R Budd, Julie L Sprague, Marvin A Dewar

**Affiliations:** 1 Department of Industrial and Systems Engineering University of Florida Gainesville, FL United States; 2 Public Health Sciences Division Fred Hutchinson Cancer Research Center Seattle, WA United States; 3 University of Florida Health Physicians Gainesville, FL United States

**Keywords:** patient portal function, user subgroup identification, heterogeneous causal effect, primary care service utilization, appointment adherence

## Abstract

**Background:**

Patient portals are now widely available and increasingly adopted by patients and providers. Despite the growing research interest in patient portal adoption, there is a lack of follow-up studies describing the following: whether patients use portals actively; how frequently they use distinct portal functions; and, consequently, what the effects of using them are, the understanding of which is paramount to maximizing the potential of patient portals to enhance care delivery.

**Objective:**

To investigate the characteristics of primary care patients using different patient portal functions and the impact of various portal usage behaviors on patients’ primary care service utilization and appointment adherence.

**Methods:**

A retrospective, observational study using a large dataset of 46,544 primary care patients from University of Florida Health was conducted. Patient portal users were defined as patients who adopted a portal, and adoption was defined as the status that a portal account was opened and kept activated during the study period. Then, users were further classified into different user subgroups based on their portal usage of messaging, laboratory, appointment, and medication functions. The intervention outcomes were the rates of primary care office visits categorized as arrived, telephone encounters, cancellations, and no-shows per quarter as the measures of primary care service utilization and appointment adherence. Generalized linear models with a panel difference-in-differences study design were then developed to estimate the rate ratios between the users and the matched nonusers of the four measurements with an observational window of up to 10 quarters after portal adoption.

**Results:**

Interestingly, a high propensity to adopt patient portals does not necessarily imply more frequent use of portals. In particular, the number of active health problems one had was significantly negatively associated with portal adoption (odds ratios [ORs] 0.57-0.86, 95% CIs 0.51-0.94, all *P*<.001) but was positively associated with portal usage (ORs 1.37-1.76, 95% CIs 1.11-2.22, all *P*≤.01). The same was true for being enrolled in Medicare for portal adoption (OR 0.47, 95% CI 0.41-0.54, *P*<.001) and message usage (OR 1.44, 95% CI 1.03-2.03, *P*=.04). On the impact of portal usage, the effects were time-dependent and specific to the user subgroup. The most salient change was the improvement in appointment adherence, and patients who used messaging and laboratory functions more often exhibited a larger reduction in no-shows compared to other user subgroups.

**Conclusions:**

Patients differ in their portal adoption and usage behaviors, and the portal usage effects are heterogeneous and dynamic. However, there exists a lack of *match* in the patient portal market where patients who benefit the most from patient portals are not active portal adopters. Our findings suggest that health care delivery planners and administrators should remove the barriers of adoption for the portal *beneficiaries;* in addition, they should incorporate the impact of portal usage into care coordination and workflow design, ultimately aligning patients’ and providers’ needs and functionalities to effectively deliver patient-centric care.

## Introduction

The patient-centric care initiative heightened the awareness of health care systems’ responsibility to provide easily accessible ways for patients to engage in their own care and become effective health care partners. Such a mission is expected to be fulfilled with patient portals, where a portal is defined as “a secure online website that allows patients to access their medical records or communicate with their health care providers” [1]. Empowered by the rapid development of health information technologies, patient portals are now widely available and increasingly adopted by patients and providers. Effective use of these portals is expected to result in improved care access, self-management, and care coordination. Furthermore, the US federal government has authorized incentive payments to physicians who demonstrated “meaningful use” of such health information systems [2]. Consequently, patient portal research has garnered growing attention; a spate of reports of portal adoption and enrollee demographics have been published over the past decade. These studies typically described individual portal deployment or analyzed national survey data, such as the Health Information National Trends Survey (HINTS), and they reported characteristics of early portal adopters [3-7]. Along with this, whether the adoption of patient portals affects health care consumption was also investigated. Health care consumption (ie, the usage of various clinical services) is closely related to care access and coordination and is, thus, an important decision factor for service operations. Understanding the impact of portals on health care consumption can facilitate the design of service systems that accommodate patients’ portal usage, leading to enhanced efficiency of service operations and improved patient access to care. However, most reviews reported mixed evidence about the effect of patient portals on health care consumption—whether portal adoption will increase or decrease outpatient office visits was debated [8-12]—and the only consensus was that patient portals were used as a complement rather than a substitute of usual clinical services [13-16]. In addition, the number of appointment no-shows has been chosen to serve as an indicator to infer patient engagement [17], and it has been reported that portal enrollment is significantly related to decreases in appointment no-shows [18-20]. However, such studies mainly captured the association but not the causation between portal enrollment and no-show reduction. It motivated us to carry out a study that could account for measurable confounders and is robust to unmeasured confounders, hence, unveiling the causal effect of portal usage. In particular, we chose to investigate primary care office visit and telephone encounter rates (per quarter) as the measures of primary care service utilization as well as appointment cancellation and no-show rates (per quarter) as the measures of appointment adherence.

Despite the growing interest in portal adoption, there is a notable paucity of follow-up studies describing whether patients use portals actively and how distinct portal functions, such as messaging, laboratory, appointment, and medication, are used after adoption. The successful achievement of the promise to improve care access, self-management, and care coordination is intrinsically linked to the extent to which portals are used. We hypothesized that a patient who actively communicates with physicians using secure messages will benefit more from adopting a patient portal than one who never uses it after adoption. This is evidenced by the literature that states that messaging usage is associated with patient engagement [21,22]. In addition to messaging, the appointment function of portals offers an alternative way to make appointments than by phone calls and makes it easier for patients to reschedule or cancel their appointments. It can be hypothesized that with more freedom to manage appointments, patients will be more adherent to their appointments. Furthermore, with the laboratory and the medication functions of portals, patients can easily access their lab results and refill prescriptions online. Reminders can be sent from portals as an intervention to encourage patients to check their test results or refill their medications. Overall, we believed the convenience brought by patient portals to patients will enable them to be the owners of their health and be actively engaged in their care management. Lastly, we hypothesized that patients’ portal usage behaviors are heterogeneous, and different portal functions might be perceived with distinct values by users with various characteristics. To test these hypotheses, it is necessary to look at how patient characteristics are associated with portal usage and how different portal usage patterns affect patients’ care consumption and adherence to appointments.

The evaluation of how often patients access portals and what they do with them was not given enough attention in the past. Ignoring such a variety behaviors of patients might lead to the misspecification of portal effects on patients. For instance, if a subgroup of patients is doing significantly better with portals, whereas another group is doing worse, aggregating them can potentially lead to a conclusion of “no change.” Furthermore, portal adoption and the subsequent usage can influence patients over time, and the time trend in portal usage effects should not be overlooked; from an operational and a strategic point of view, both short-term and long-term impacts matter. Therefore, instead of solely relying on the observations from cross-sectional data, we sought to examine longitudinal data and focused on not only portal adoption but also on portal usage, aiming to investigate the following: (1) the characteristics of people who are more likely to adopt a patient portal, (2) among patients who have adopted portals, determine who uses portals more often and the characteristics of people associated with different portal function usage behaviors, and (3) whether the primary care service utilization and appointment adherence of patients who have adopted portals are affected by their different portal usage behaviors, featuring both the amount of use and the type of portal functions used. The answers to these questions are vital to informing (1) the design and implementation of patient portals, (2) the service system operations, such as the daily workflow design, and (3) the policy guidelines, such as remuneration models to compensate providers’ portal time.

## Methods

### Study Setting

#### Data Source

This study used the data generated by a large primary care patient cohort affiliated with the University of Florida (UF) Health; the data protocol was approved by the UF Institutional Review Board. In 2011, UF Health started offering an electronic patient portal named *MyUFHealth*, or *MyChart*, which allows patients to access to portions of their medical records (eg, released test results and after-visit summaries), communicate with the clinical service providers using secure messaging, request prescription refills, and manage outpatient appointments. Monthly clinical service utilization and portal activities of individual patients were generally not frequent; therefore, the time unit used in this study was one quarter: January-March (first quarter, Q1), April-June (second quarter, Q2), July-September (third quarter, Q3), and October-December (fourth quarter, Q4). For instance, Y13 Q3 stands for the third quarter of the year 2013. The study period was from July 1, 2013, to June 30, 2016. During the study period, there were 46,544 UF Health patients who had at least one visit to UF Health family medicine clinics. More than 95% of them came from North Central Florida.

#### Study Sample

Because the portal accounts of patients under 18 years old are typically managed by their legal guardians, we restricted our analysis to adult patients. We further restricted the study to insured patients who (1) chose UF Health as their primary health care provider, (2) enrolled in UF Health before the start of the study period, and (3) maintained an enrollment status until the end of the study period. As such, their primary care service utilization within the UF Health network can be fully captured. It is worth noting that UF Health is the leading care provider in the study region, and primary care services rendered to insured patients outside of the UF Health network are very limited. In addition, to ensure a contrast of before-after portal adoption and to capture the portal usage effects over time, we defined users in our study as patients who adopted *MyUFHealth* during periods Y13 Q4 to Y15 Q3. We excluded patients who (1) adopted the portal before the study period, (2) adopted the portal relatively recently (ie, adopted the portal in Y15 Q4 or after), or (3) were temporary users (ie, adopted the portal but closed their accounts before the study ended). These inclusion and exclusion criteria led to 17,580 nonusers and 4312 users.

#### Variables and Measures

Patients’ demographic and socioeconomic information, including age category, gender, race or ethnicity, marital status, insurance type, and their active problem number (APN), were obtained from their electronic medical records (EMRs). The APN is the number of problems in a patient’s active problem list, which captures patients’ chronic conditions and any ongoing impactful conditions that are resolvable but are important for physicians to be aware of to make clinical decisions. Notably, an ailment like a common cold or flu does not appear in the active problem list, and this list is typically reviewed at each patient encounter and updated—adding or deleting problems—whenever deemed necessary. Accordingly, a patient’s APN is considered as a time-varying confounder to account for individual disease burdens. It should also be noted that patients tend to use care services intensively right after an onset diagnosis of a new health condition and less frequently later, due to the resolution of the triggering health care condition [8]. A patient’s disease process (ie, an onset of a condition, followed by an episode of treatment, possibly including the resolution of the condition) can be nested within the process of portal adoption and subsequent usage. Therefore, we proposed a study design that controls for the time a new diagnosis was made (ie, when a visit type coded as *new* appeared in the EMR), allowing an assessment of the natural disease process.

To characterize portal usage patterns, we focused on four major portal functions that are regularly accessed by users: messaging (MESG), laboratory (LAB), medication (MED), and appointment (APPT). Patient portal usage is measured by the amount of use per quarter by function type. In particular, variable *MESG_it_* is defined as the count of messaging-related activities by user *i* at quarter *t*, such as *open a message box*, *read a message*, *delete a message*, and *send a message* by patients. Variables *LAB_it_*, *MED_it_*, and *APPT_it_* represent the count of actions related to laboratory activities (eg, check lab test results and request lab test); actions related to medication, such as check medication list and request drug or prescription refill; as well as actions related to appointments, such as appointment scheduling, appointment status checking, and cancel or reschedule an appointment, respectively.

To evaluate how portal usage affects primary care service utilization and appointment adherence, rates of office visits categorized as *arrived*, *cancelled*, or *no-show*, as well as *telephone encounters* per quarter were measured. Patients’ office visits and telephone encounters within the UF Health network were used as an indicator of their overall primary care service utilization.

### User Subgroup Clustering

Patients’ portal activities differ across individuals and time: they might use a specific portal function more or less frequently based on their intrinsic preferences or immediate care needs, which might change with time. Therefore, we considered the portal usage over the course of a postadoption phase as the exposure and the use of primary care services as the outcome. We aimed to investigate the causality by examining the time dynamic behaviors in both exposures and outcomes. To characterize the time-varying exposures, we categorized patients into user subgroups and investigated the makeup of each subgroup, as well as the portal activity features associated with each subgroup.

Specifically, to cluster patients, we defined an activity feature vector (*MESG_it_,LAB_it_,MED_it_,APPT_it_*) (ie, the amount of function usage by user *i* at *t* quarters postadoption). This entails a pattern recognition problem with each user *i* being characterized by a set of ordered vectors 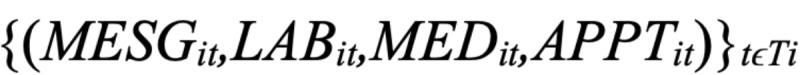, where *T_i_* is the set of observation times postadoption for user *i*. A naive treatment is to take the average utilization over time and create a compact feature vector. However, this cannot separate the cases where a patient was moderate in messaging utilization in each quarter, versus a patient who did not use messaging except for one quarter of intensive use that brings the average utilization into the moderate level. Therefore, we proposed a two-stage clustering method to mitigate the *flaw of averages* and allow some assessment of the longitudinal usage patterns.

In the first stage, we characterized the relationships between four functions, for instance, whether there were two or more functions that were frequently used together at any time. The spherical clustering method [23] was employed to cluster activity feature vectors (*MESG_it_,LAB_it_,MED_it_,APPT_it_*). The difference in scales can be addressed by this method. For instance, the overall usage of messaging is one order of magnitude higher than that of medication. As a result, five activity clusters—*C_MESG_, C_LAB_, C_MED_, C_APPT_*, and *C_M&L_*—were identified, which were named after their dominant activities. For instance, if patient *i* used messaging many times but not so much for the rest of the functions at time *t*, the activity feature vector will then be labeled with “*C_MESG_*” at time *t*. For activity feature vectors with MESG and LAB functions used together and more often than others, a label of “*C_M&L_*” was assigned. In addition, a sixth cluster named *C_Silent_* was assigned for any activity feature vector being a vector of zeros. After the label assignment, a patient was then characterized by a |*T_i_*|-dimensional pattern vector, *(C_i1_, C_i2_,..., C_i|Ti|_* ), where 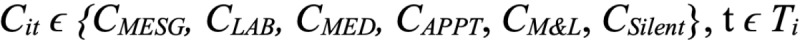. For example (*C_MESG_, C_Silent,_ C_MESG_, C_Silent,_ C_Silent_)* is a labeled pattern vector for a patient with five observations postadoption (ie, *T_i_*=5). Based on analyzing the data, the order of labels was quite random and, thus, was not featured into user types.

In the second stage, each user was assigned to one user type based on the number of occurrences of various activity clusters (ie, labels) over the postadoption period. For instance, with the above sample patient, cluster *C_MESG_* has a frequency of 2/5, cluster *C_Silent_* has a frequency of 3/5, and the frequency is 0 for the rest of the clusters. A user feature vector (2/5,0,0,0,0,3/5) representing the frequencies of belonging to clusters *{C_MESG_, C_LAB_, C_MED_, C_APPT_*, *C_M&L_*, *C_Silent_*} was created for the patient-level clustering. Because the user feature vector was already normalized, the K-means with Euclidian distance clustering method was used. As a result, five clusters were identified to represent five user types—*U_MESG_, U_LAB_, U_APPT_*, *U_M&L_*, and *U_Silent_*—named after their dominant activity clusters. For instance, *U_Silent_* represents the type of users who were, in general, inactive postadoption.

To summarize, we created activity feature vectors and collected activity feature vectors of all patients at every quarter after portal adoption to find common patterns, referred to here as activity clusters. This concluded the first-stage clustering. We then labeled each activity feature vector—per patient per time—with a membership (ie, belonging to one out of six activity clusters). This yielded a longitudinal activity pattern vector for each patient. Notably, such pattern vectors were of different dimensions, due to different observational time lengths postadoption. Thus, they were further mapped to a user feature vector with a fixed dimension of six for each patient. The user feature vectors of all patients were then clustered, which led to the final five user subgroups. This concluded the second-stage clustering. The illustration of the two-stage clustering and the descriptive statistics of the clustering results can be found in Multimedia Appendix 1.

### Vector Matching Using Propensity Scores

Confounders are the determinants of exposure that are associated with outcomes (ie, variables that potentially affect both outcomes), for example, primary care service utilization, and exposure to different types of interventions (ie, becoming a specific type of user). To reduce the bias in causal inference due to confounders, we matched the users and nonusers using the vector matching method [24]. We first calculated propensity scores by estimating the probability of belonging to a user subgroup 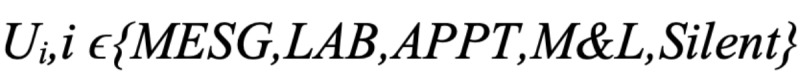 using multinomial logit regression (MLR). The covariates of the MLR model include the time-invariant characteristics (ie, age category, gender, race, marital status, and insurance type) and the time-varying variables (ie, APN and primary care office visits categorized as *arrived*, *no-show*, or *cancellation*, as well as *telephone encounters*) measured at their baseline. Notably, only the most recent marital status was recorded in the system. In addition, insurance types can change over time; however, the change was infrequent in our patient population, due to a relatively limited study time span. Therefore, a patient’s insurance type was treated as a time-invariant variable. The baseline values are observations averaged over the time period before adoption for users, and before Y14 Q4 for nonusers. The matching was based on the propensity score vectors obtained from MLR. A nonuser was matched to a user in one subgroup with a similar propensity score vector. To enhance the matching outcome, we further allowed nonusers to be exactly matched to users upon having multiple candidates available in the same propensity score stratum.

### Generalized Linear Model for Heterogeneous Portal Usage Effects

In addition to matching patient demographics, the time-varying disease burden and the dynamic disease process needed to be addressed, which motivated a causal inference study accounting for both time-invariant and time-varying confounders. A panel difference-in-differences (DID) framework using generalized linear models was developed. The framework was similar to that in Zhong et al [25] but was generalized to capture heterogeneous portal usage effects. The detail of the model can be found in Multimedia Appendix 1.

Using such a framework, one can estimate the rate ratios (RRs) between the users and the matched nonusers for the targeted outcomes, including rates (per quarter) of office visits categorized as *arrived*, *cancelled*, or *no-show*, as well as *telephone encounters*. RRs for different user subgroups after portal adoption with an observational window of up to 10 quarters were obtained. An RR being significantly greater than 1 at a given quarter postadoption implies that the corresponding rate (eg, office visit rate) of the users was significantly larger than that of the nonusers at that quarter, which measures the time-dependent portal effects. In this study, all statistical analyses were performed using R, version 3.3.1 (The R Foundation), with two-sided statistical tests at a .05 significance level.

## Results

### Patient Portal Adoption

A logistic regression model was built to predict portal adoption; the odds ratios (ORs) obtained are exhibited in Table 1. The following were negatively associated with portal adoption: Hispanic and black or African American race versus white (OR 0.38 vs 0.53, 95% CI 0.19-0.69 vs 0.69-0.92, *P*=.003 vs *P*<.001); male gender (OR 0.64, 95% CI 0.59-0.68, *P*<.001); marital status as *not married* (ie, single, divorced, widowed, or not having a life partner or a significant other) in contrast to *married* (ORs 0.30-0.72, 95% CIs 0.22-0.83, all *P*<.001); and insurance type as not being Blue Cross Blue Shield (ORs 0.23-0.73, 95% CIs 0.12-0.80, all *P*<.001). Moreover, a high baseline APN (ORs 0.57 and 0.86, 95% CIs 0.51-0.63 and 0.80-0.94, all *P*<.001) and a high baseline no-show rate (OR 0.29, 95% CI 0.21-0.40, *P*<.001) were negatively associated with portal adoption. The following were positively associated with portal adoption: being above 30 years of age in contrast to being 19-30 years of age (ORs 1.20-1.28, 95% CIs 1.07-1.52, all *P*<.01) and having a high baseline telephone encounter rate (OR 1.13, 95% CI 1.06-1.19, *P*<.001).

### Patient Portal Usage

Portal users’ usage summary statistics are presented here. The mean portal log-in rate was 6.85 per user per quarter (SD 12.11) with a median of 3 (IQR 8). The most frequently used portal function (per quarter) was messaging (mean 17.67, SD 34.69; median 5, IQR 20), followed by laboratory (mean 12.22, SD 28.04; median 1, IQR 13), appointment (mean 7.65, SD 19.89; median 1, IQR 7), and medication (mean 1.73, SD 4.05; median 0, IQR 2). The average number of secure messages sent from patients—we counted unique conversation threads, which can include multiple back-and-forth messages—was 1.07 per quarter. It was observed that 1214 users were very active and constantly accessed the portal postadoption. The remaining users did not use the portal in at least one quarter after adopting it.

**Table 1:** Odds ratios (ORs) of patient characteristics for portal adoption.

Patient characteristics		OR (95% CI) (users versus nonusers)	*P* value
**Age in years (reference: 19-30)**			
	31-45	1.22 (1.09-1.36)	<.001
	46-64	1.20 (1.07-1.34)	.002
	65+	1.28 (1.07-1.52)	.007
**Gender (reference: female)**			
	Male	0.64 (0.59-0.68)	<.001
**Race (reference: white)**			
	Asian	1.17 (0.96-1.42)	.11
	Black or African American	0.53 (0.48-0.58)	<.001
	Hispanic	0.38 (0.19-0.69)	.003
	Others	0.80 (0.69-0.92)	.002
**Marital status (reference: married or companion)**			
	Divorced or separated	0.72 (0.61-0.83)	<.001
	Other	0.30 (0.22-0.39)	<.001
	Single	0.66 (0.60-0.71)	<.001
	Widowed	0.50 (0.40-0.62)	<.001
**Insurance type (reference: Blue Cross Blue Shield^a^)**			
	Commercial or managed care	0.73 (0.66-0.80)	<.001
	Medicaid	0.42 (0.36-0.48)	<.001
	Medicare	0.47 (0.41-0.54)	<.001
	Other	0.23 (0.12-0.40)	<.001
	Self-pay	0.46 (0.39-0.55)	<.001
**Baseline care service utilization (continuous)**			
	Telephone encounter	1.13 (1.06-1.19)	<.001
	Office visit: arrived	0.97 (0.90-1.04)	.41
	Office visit: cancelled	1.06 (0.94-1.19)	.36
	Office visit: no-show	0.29 (0.21-0.40)	<.001
**Baseline APN^b^ (reference: ≤2.5)**			
	>2.5 and ≤7	0.86 (0.80-0.94)	<.001
	>7	0.57 (0.51-0.63)	<.001

^a^Blue Cross Blue Shield is a type of commercial insurance with a sufficiently large body of enrollees that can enable us to statistically identify its effect.

^b^APN: active problem number.

### Patient User Subgroups

After the two-stage clustering, we identified 615, 663, 1006, 536, and 1492 patients in user subgroups, *U_LAB_* (14.3%), *U_M&L_* (15.4%), *U_MESG_* (23.3%), *U_APPT_* (12.4%), and *U_Silent_* (34.6%), respectively. The association between user types and patient characteristics was analyzed using MLR. Patients’ baseline care service utilization and marital status were not significantly associated with user types (*P*>.05). Married people or people with a life partner or a significant other, although being more likely to adopt portals compared to single people, were not less likely to be *U_Silent_*. The ORs of the significantly relevant patient characteristics obtained from the MLR model are shown in Table 2. It can be seen that age is the most important predictor of user types. The ORs of using appointment functions strictly decrease with age (31-45, 46-64, and 65+ years: ORs 0.52, 0.34, and 0.19, respectively, 95% CIs 0.11-0.67, all *P*<.001). On the contrary, the intention of using messaging increases with age (31-45 years: OR 0.99, 95% CI 0.76-1.29, *P*>.05; 46-64 and 65+ years: ORs 1.38 and 1.50, 95% CIs 1.07-1.78 and 1.00-2.23, *P*=.01 and .04, respectively). Regarding gender, males used the laboratory function less often, such as being type *U_M&L_* (OR 0.61, 95% CI 0.50-0.76, *P*<.001) and being type *U_LAB_* (OR 0.75, 95% CI 0.61-0.93, *P*=.01) in contrast to being silent. In addition, male users were more inactive: no ORs were significantly larger than 1. On race and ethnicity, compared to white users, Asian users used the messaging less (OR 0.44, 95% CI 0.26-0.73, *P*=.002) but used the laboratory more (OR 1.58, 95% CI 1.04-2.39, *P*=.03). Black or African American users made significantly more appointments via the portal (OR 1.36, 95% CI 1.05-1.76, *P*=.02). Hispanic users were relatively silent: ORs were insignificant due to a small sample size.

On insurance type, it is interesting to note that although Medicaid and Medicare patients tended not to adopt a portal compared to Blue Cross Blue Shield patients, Medicaid patients shared a similar user type distribution as Blue Cross Blue Shield patients (for all ORs, *P*>.05). Moreover, Medicare patients used messaging significantly more (OR 1.44, 95% CI 1.03-2.03, *P*=.04) and were less inactive compared to other insurance types: no ORs were significantly smaller than 1. It should be noted that Medicare patients also include those who are less than 65 years of age but have received Social Security Disability Insurance checks for at least 24 months or have been diagnosed with end-stage renal disease [26]. In our patient population, we have around 28% of Medicare patients who were less than 65 years of age.

Lastly, a heavy disease burden in contrast to a small APN was significantly positively associated with frequent portal usage of any activity types (ORs 1.37-1.76, 95% CIs 1.11-2.22, all *P*≤.01), which is contrary to the observation that patients with a heavy disease burden tended not to adopt a portal. To demonstrate the quality of matching, the characteristics of the users and the nonusers before and after matching are shown in Table 3.

**Table 2:** Odds ratios (ORs) of patient characteristics for being in different user subgroups.

Patient characteristics		Nonsilent versus silent users							
User types		LAB^a^, OR (95% CI)	*P* value	MESG^b^ and LAB, OR (95% CI)	*P* value	MESG, OR (95% CI)	*P* value	APPT^c^, OR (95% CI)	*P* value
**Age categories (years) (reference: 19-30)**									
	31-45	0.76 (0.58-1.00)	.05	0.85 (0.64-1.13)	.25	0.99 (0.76-1.29)	.92	0.52 (0.40-0.67)	<.001
	46-64	0.83 (0.63-1.09)	.18	1.11 (0.84-1.47)	.45	1.38 (1.07-1.78)	.01	0.34 (0.25-0.45)	<.001
	65+	0.77 (0.48-1.25)	.29	1.01 (0.64-1.59)	.98	1.50 (1.01-2.23)	.04	0.19 (0.11-0.33)	<.001
**Gender (reference: female)**									
	Male	0.75 (0.61-0.93)	.01	0.61 (0.50-0.76)	<.001	0.95 (0.80-1.13)	.57	1.13 (0.91-1.41)	.26
**Race (reference: white)**									
	Asian	1.58 (1.04-2.39)	.03	0.86 (0.54-1.39)	.55	0.44 (0.26-0.73)	.002	0.54 (0.29-1.00)	.05
	Black or African American	1.18 (0.91-1.53)	.22	0.84 (0.64-1.10)	.20	0.90 (0.71-1.14)	.38	1.36 (1.05-1.76)	.02
	Hispanic	0.75 (0.15-3.66)	.72	0.36 (0.04-2.99)	.35	0.29 (0.04-2.36)	.25	N/A^d^	.94
	Other	1.29 (0.90-1.86)	.17	0.90 (0.61-1.33)	.60	0.89 (0.63-1.25)	.49	0.95 (0.63-1.42)	.79
**Insurance (reference: Blue Cross Blue Shield)**									
	Commercial or managed care	0.82 (0.64-1.06)	.13	0.70 (0.54-0.90)	.01	1.04 (0.84-1.29)	.71	0.62 (0.47-0.82)	<.001
	Medicaid	1.09 (0.74-1.60)	.65	1.00 (0.68-1.47)	.99	1.10 (0.77-1.57)	.59	0.88 (0.60-1.31)	.54
	Medicare	1.27 (0.83-1.94)	.27	1.25 (0.84-1.87)	.28	1.44 (1.03-2.03)	.04	1.43 (0.90-2.29)	.13
	Other	0.35 (0.04-2.86)	.33	0.54 (0.11-2.63)	.45	0.20 (0.02-1.68)	.14	0.45 (0.05-3.70)	.45
	Self-pay	0.53 (0.30-0.94)	.03	0.56 (0.33-0.96)	.03	1.00 (0.66-1.51)	>.99	0.87 (0.53-1.43)	.58
**Baseline APN^e^** **(reference: ≤2.5)**									
	>2.5 and ≤7	1.56 (1.26-1.94)	<.001	1.37 (1.11-1.70)	.004	1.37 (1.13-1.65)	.001	1.45 (1.15-1.82)	.001
	>7	1.52 (1.14-2.01)	.004	1.66 (1.27-2.16)	<.001	1.76 (1.40-2.22)	<.001	1.53 (1.13-2.08)	.01

^a^LAB: laboratory.

^b^MESG: messaging.

^c^APPT: appointment.

^d^Not applicable—the CI cannot be estimated due to the small sample size.

^e^APN: active problem number.

**Table 3:** Patient characteristics of unmatched nonusers, users, and matched nonusers.

Characteristics		Unmatched nonusers (N=17,580), n (%)	Users (N=4024), n (%)	*P* value	Matched nonusers (N=4024), n (%)	*P* value
**Age categories (years)**				**<.001**		**.53**
	19-30	3777 (21.48)	769 (19.11)		803 (19.96)	
	31-45	4220 (24.08)	1152 (28.63)		1181 (29.35)	
	46-64	5662 (32.21)	1418 (35.24)		1364 (33.90)	
	65+	3921 (22.30)	685 (17.02)		676 (16.80)	
**Gender**				**<.001**		**.39**
	Female	10,443 (59.40)	2648 (65.81)		2610 (64.86)	
	Male	7137 (40.60)	1376 (34.19)		1414 (35.14)	
**Race**				**<.001**		**>.99**
	Asian	350 (1.99)	119 (2.96)		119 (2.96)	
	Black or African American	5509 (31.34)	675 (16.77)		675 (16.77)	
	Hispanic	91 (0.52)	9 (0.22)		9 (0.22)	
	Other	1035 (5.89)	241 (5.99)		241 (5.99)	
	White	10,595 (60.27)	2980 (74.06)		2980 (74.06)	
**Marital status**				**<.001**		**.62**
	Divorced or separated	1372 (7.80)	222 (5.52)		231 (5.74)	
	Married or companion	6820 (38.79)	2197 (54.60)		2196 (54.57)	
	Other	472 (2.68)	53 (1.32)		41 (1.02)	
	Single	8054 (45.81)	1461 (36.31)		1477 (36.70)	
	Widowed	862 (4.90)	91 (2.26)		79 (1.96)	
**Insurance**				**<.001**		**.81**
	Blue Cross Blue Shield	5986 (34.05)	2122 (52.73)		2114 (52.53)	
	Commercial or managed care	2797 (15.91)	742 (18.44)		755 (18.76)	
	Medicaid	2683 (15.26)	278 (6.91)		257 (6.39)	
	Medicare	4906 (27.91)	718 (17.84)		726 (18.04)	
	Other	145 (0.82)	11 (0.27)		17 (0.42)	
	Self-pay	1063 (6.05)	153 (3.80)		155 (3.85)	
**Baseline APN^a^**				**<.001**		**.71**
	≥0 and ≤2.5	5523 (31.42)	1704 (42.35)		1704 (42.35)	
	>2.5 and ≤7	6090 (34.64)	1482 (36.83)		1509 (37.50)	
	>7	5967 (33.94)	838 (20.83)		811 (20.15)	

^a^APN: active problem number.

### Primary Care Service Utilization and Appointment Adherence

#### Overview

**Figure 1:**
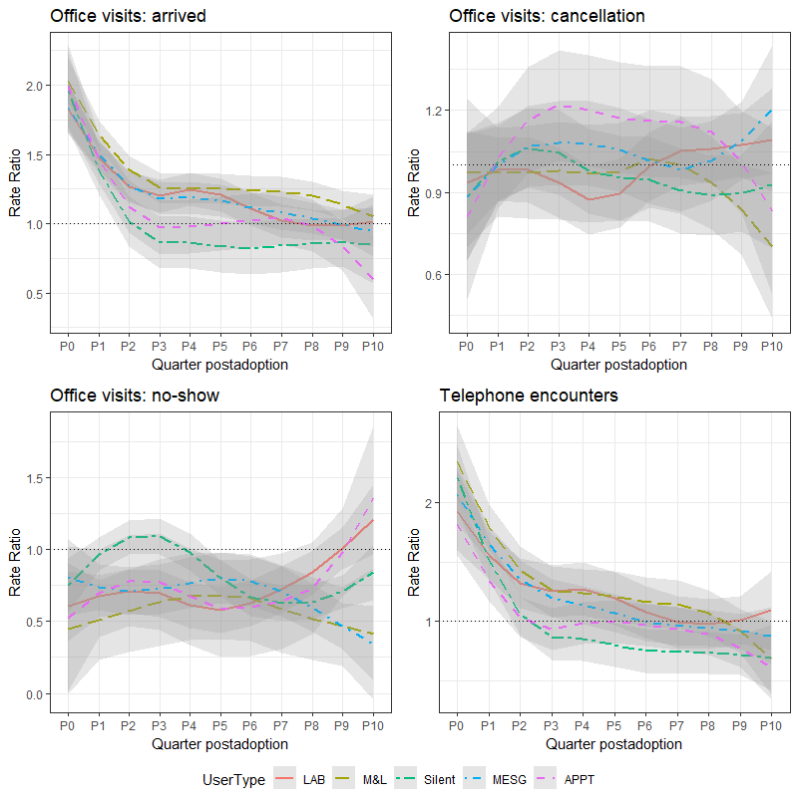
Quarterly rate ratios (users/nonusers) of primary care office visits categorized as *arrived, cancellation, no-show,* as well as *telephone encounters* postadoption of the portal. APPT: appointment; LAB: laboratory; MESG: messaging; M&L: messaging and laboratory; Silent: being inactive.

Using the panel DID models (see Multimedia Appendix 1), we compared the utilization of primary care services between the matched nonusers and different user subgroups before and after portal adoption. The RRs of the users to the nonusers attributable to portal usage effects at each quarter postadoption are shown in Figure 1, and the corresponding RRs can be found in Table 4. The RRs measure the time-varying difference between the portal users and the matched nonusers at each quarter after portal adoption. To interpret, an RR being 1 suggests that there is no difference between the users and the matched nonusers, and thus there is no significant portal effect.

**Table 4:** Quarterly rate ratios (RRs) between the portal users and the matched nonusers of office visits categorized as arrived, telephone encounter, cancellation, and no-show for different user subgroups after portal adoption.

User type and period^a^		Arrived			Telephone encounter		Cancellation		No-show	
		RR (95% CI)	*P* value	RR (95% CI)		*P* value	RR (95% CI)	*P* value	RR (95% CI)	*P* value
**LAB^b^**										
	P0	1.91 (1.72-2.11)	<.001	2.07 (1.78-2.37)		<.001	0.89 (0.71-1.08)	.27	0.60 (0.36-0.83)	.001
	P1	1.36 (1.20-1.52)	<.001	1.29 (1.06-1.51)		.004	1.04 (0.80-1.27)	.78	0.71 (0.42-1.01)	.06
	P2	1.17 (1.02-1.32)	.02	1.28 (1.06-1.50)		.01	1.05 (0.79-1.31)	.70	0.67 (0.37-0.97)	.03
	P3	1.23 (1.07-1.39)	.002	1.36 (1.13-1.60)		<.001	0.99 (0.74-1.23)	.91	0.71 (0.40-1.02)	.07
	P4	1.27 (1.10-1.44)	<.001	1.21 (0.97-1.44)		.06	0.75 (0.54-0.97)	.03	0.69 (0.37-1.00)	.05
	P5	1.25 (1.07-1.43)	.003	1.25 (0.99-1.50)		.04	0.92 (0.65-1.19)	.58	0.49 (0.21-0.78)	.001
	P6	1.08 (0.89-1.27)	.39	1.09 (0.83-1.35)		.49	1.02 (0.69-1.36)	.89	0.55 (0.19-0.92)	.02
	P7	0.99 (0.80-1.18)	.93	0.96 (0.70-1.22)		.77	1.06 (0.68-1.44)	.76	0.90 (0.36-1.44)	.72
	P8	1.00 (0.78-1.22)	>.99	0.93 (0.63-1.23)		.67	1.07 (0.62-1.52)	.77	0.75 (0.17-1.33)	.40
	P9	1.02 (0.76-1.28)	.88	1.09 (0.72-1.45)		.63	0.97 (0.50-1.44)	.90	0.96 (0.21-1.70)	.91
	P10	1.00 (0.69-1.31)	.98	1.06 (0.60-1.52)		.79	1.15 (0.48-1.81)	.67	1.25 (0.18-2.32)	.65
**MESG^c^ and LAB**										
	P0	2.07 (1.88-2.26)	<.001	2.42 (2.12-2.72)		<.001	0.97 (0.79-1.15)	.77	0.42 (0.23-0.61)	<.001
	P1	1.59 (1.43-1.76)	<.001	1.67 (1.43-1.91)		<.001	0.94 (0.75-1.14)	.57	0.60 (0.36-0.85)	.002
	P2	1.36 (1.20-1.51)	<.001	1.39 (1.18-1.61)		<.001	1.06 (0.83-1.29)	.60	0.49 (0.26-0.72)	<.001
	P3	1.20 (1.05-1.34)	.004	1.17 (0.97-1.37)		.08	0.90 (0.68-1.13)	.40	0.60 (0.31-0.89)	.01
	P4	1.36 (1.20-1.52)	<.001	1.43 (1.19-1.66)		<.001	0.96 (0.73-1.18)	.70	0.69 (0.39-1.00)	.05
	P5	1.21 (1.06-1.37)	.004	1.08 (0.87-1.29)		.43	1.08 (0.82-1.35)	.54	0.84 (0.47-1.21)	.39
	P6	1.26 (1.08-1.43)	.001	1.24 (1.00-1.48)		.03	0.86 (0.62-1.10)	.25	0.35 (0.11-0.59)	<.001
	P7	1.19 (1.01-1.37)	.03	1.07 (0.83-1.31)		.55	1.12 (0.81-1.44)	.44	0.86 (0.43-1.29)	.53
	P8	1.28 (1.08-1.48)	.002	1.15 (0.88-1.42)		.24	0.97 (0.67-1.27)	.82	0.45 (0.13-0.77)	.001
	P9	1.12 (0.91-1.33)	.25	0.97 (0.70-1.25)		.85	0.73 (0.43-1.03)	.08	0.37 (0.02-0.71)	<.001
	P10	1.05 (0.79-1.30)	.72	0.65 (0.36-0.93)		.05	0.75 (0.35-1.15)	.22	0.47 (0.03-0.97)	.04
**MESG**										
	P0	1.91 (1.75-2.06)	<.001	2.14 (1.90-2.38)		<.001	0.83 (0.69-0.97)	.02	0.85 (0.60-1.10)	.24
	P1	1.40 (1.27-1.53)	<.001	1.50 (1.31-1.69)		<.001	1.08 (0.89-1.27)	.42	0.69 (0.44-0.94)	.02
	P2	1.18 (1.06-1.30)	.001	1.37 (1.20-1.55)		<.001	1.14 (0.92-1.35)	.21	0.60 (0.36-0.84)	.001
	P3	1.24 (1.12-1.36)	<.001	1.16 (1.00-1.32)		.04	1.04 (0.84-1.24)	.69	0.82 (0.54-1.11)	.22
	P4	1.22 (1.09-1.34)	<.001	1.21 (1.04-1.39)		.01	0.99 (0.79-1.19)	.94	0.80 (0.51-1.09)	.18
	P5	1.12 (1.00-1.24)	.04	1.00 (0.84-1.16)		>.99	1.20 (0.96-1.44)	.11	0.65 (0.39-0.92)	.01
	P6	1.17 (1.04-1.30)	.01	1.08 (0.91-1.25)		.32	0.99 (0.78-1.20)	.94	0.92 (0.59-1.25)	.62
	P7	1.04 (0.92-1.17)	.50	0.86 (0.70-1.02)		.11	0.88 (0.66-1.09)	.27	0.72 (0.41-1.03)	.08
	P8	1.03 (0.90-1.16)	.65	0.99 (0.81-1.18)		.94	1.09 (0.82-1.36)	.53	0.51 (0.23-0.79)	.001
	P9	1.03 (0.89-1.18)	.66	0.98 (0.78-1.17)		.83	1.12 (0.82-1.42)	.43	0.42 (0.15-0.69)	<.001
	P10	0.93 (0.76-1.09)	.39	0.84 (0.62-1.06)		.20	1.18 (0.79-1.57)	.37	0.39 (0.07-0.71)	<.001
**APPT^d^**										
	P0	2.10 (1.88-2.32)	<.001	1.93 (1.62-2.24)		<.001	0.76 (0.59-0.93)	.01	0.47 (0.28-0.67)	<.001
	P1	1.29 (1.12-1.46)	<.001	1.14 (0.91-1.36)		.20	1.15 (0.87-1.42)	.30	0.82 (0.49-1.14)	.27
	P2	1.08 (0.92-1.23)	.31	1.00 (0.79-1.21)		.98	1.14 (0.84-1.43)	.38	0.70 (0.39-1.02)	.06
	P3	1.00 (0.84-1.15)	.95	0.96 (0.74-1.18)		.73	1.22 (0.87-1.57)	.21	0.79 (0.41-1.17)	.29
	P4	1.06 (0.88-1.23)	.51	0.99 (0.76-1.23)		.95	1.16 (0.81-1.51)	.37	0.78 (0.40-1.16)	.26
	P5	0.87 (0.71-1.03)	.14	1.02 (0.77-1.27)		.89	1.30 (0.87-1.72)	.17	0.48 (0.15-0.80)	.002
	P6	1.15 (0.94-1.36)	.13	0.98 (0.71-1.24)		.88	0.98 (0.64-1.31)	.88	0.56 (0.22-0.90)	.01
	P7	0.97 (0.76-1.18)	.77	0.91 (0.63-1.20)		.56	1.29 (0.80-1.77)	.25	0.72 (0.24-1.20)	.25
	P8	1.02 (0.78-1.26)	.88	0.86 (0.55-1.17)		.41	1.08 (0.61-1.54)	.75	0.88 (0.30-1.45)	.68
	P9	0.90 (0.64-1.16)	.47	0.92 (0.56-1.28)		.68	1.13 (0.55-1.71)	.66	0.56 (0.01-1.11)	.12
	P10	0.57 (0.30-0.83)	.02	0.54 (0.17-0.91)		.08	0.77 (0.01-1.55)	.57	1.55 (0.08-3.18)	.51
**Silent^e^**										
	P0	2.10 (1.95-2.25)	<.001	2.35 (2.11-2.58)		<.001	0.83 (0.71-0.96)	.01	0.75 (0.56-0.95)	.01
	P1	1.13 (1.03-1.24)	.01	1.29 (1.13-1.46)		<.001	1.05 (0.86-1.24)	.59	1.00 (0.69-1.30)	.98
	P2	0.97 (0.87-1.07)	.55	0.95 (0.81-1.09)		.47	1.24 (1.01-1.46)	.04	0.99 (0.68-1.31)	.96
	P3	0.89 (0.80-0.99)	.03	0.93 (0.79-1.06)		.31	0.91 (0.72-1.10)	.36	1.24 (0.87-1.62)	.21
	P4	0.92 (0.82-1.01)	.10	0.89 (0.76-1.02)		.13	0.97 (0.77-1.17)	.75	0.92 (0.61-1.23)	.61
	P5	0.80 (0.71-0.89)	<.001	0.80 (0.67-0.93)		.009	1.02 (0.80-1.24)	.85	0.80 (0.51-1.10)	.19
	P6	0.83 (0.74-0.92)	.001	0.72 (0.60-0.84)		<.001	0.92 (0.72-1.12)	.45	0.65 (0.40-0.91)	.01
	P7	0.83 (0.73-0.93)	.001	0.75 (0.62-0.88)		.001	0.89 (0.68-1.09)	.28	0.61 (0.35-0.87)	.003
	P8	0.88 (0.77-0.98)	.04	0.76 (0.61-0.90)		.003	0.92 (0.70-1.14)	.48	0.69 (0.40-0.98)	.04
	P9	0.90 (0.78-1.02)	.11	0.73 (0.58-0.88)		.003	0.86 (0.62-1.09)	.23	0.66 (0.35-0.98)	.04
	P10	0.83 (0.69-0.97)	.03	0.68 (0.50-0.86)		.004	0.94 (0.63-1.26)	.73	0.85 (0.41-1.29)	.52

^a^P0 is the time of portal adoption and P1-P10 stand for quarters 1-10 postadoption.

^b^LAB: laboratory.

^c^MESG: messaging.

^d^APPT: appointment.

^e^Silent: being inactive.

#### Office Visits

For all the user subgroups, the office visit RRs were significantly larger than 1 within 6 months after portal adoption (RRs 1.13-2.1, 95% CIs 1.03-2.25, all *P*<.001) but were decreasing over time. The difference in office visit rates between the nonusers and most users—except for silent users—2 years postadoption was not significant. For the silent users, their office visit rates were not changed or were slightly lower by around 10% after 6 months postadoption. Patients who frequently used both messaging and laboratory functions had the largest RRs of office visits categorized as *arrived*, with an approximate 20% increase. Factoring in the APN of different user subgroups, for patients with fewer active health problems, their primary care service utilization was significantly lower after portal adoption. Meanwhile, with a heavy disease burden, the utilization was temporarily increased but was not significantly changed after 2 years postadoption.

#### Telephone Encounters

The change of telephone encounters was similar to that of office visits. Telephone encounters increased significantly at the time patients adopted portals (RRs 1.93-2.42, 95% CIs 1.62-2.72, all *P*<.001) and the RRs decreased over time. The silent users’ telephone encounters were significantly lower by 20% or more after 1 year postadoption (RRs 0.68-0.80, 95% CIs 0.50-0.93, all *P*<.01).

#### Appointment Cancellation

For all user subgroups, their cancellation rates were not significantly different to the nonusers and there was no trend in cancellation RRs over time.

#### Appointment No-Show

The no-show rates were significantly lower in most quarters postadoption: that of users were lower by 30% on average than nonusers and were not changed in the remaining quarters. In particular, patients using more *messaging* and *messaging and laboratory* combined had a larger reduction in no-show rates (average RR 0.61, minimum RR 0.35, *P*<.001). In summary, using patient portals is effective in reducing no-shows, but the relationship between portal usage and primary care service utilization is more complex than the simple substitution of online for in-person care.

### Sensitivity Analysis

We analyzed the robustness of the results by changing the seeds used for conducting user subgroup clustering. This resulted in changes in user subgroup memberships: about 4.6% (95% CI 4.3-4.8) of patients had different memberships compared to the original clustering. The corresponding results were similar to the original model with respect to the overall RR trends for different outcome measures. The RR estimates and significance were slightly different but did not affect the major conclusions, which validates the robustness of this framework.

## Discussion

### Principal Findings

#### Patient Portal Adoption

In terms of the characteristics of portal adopters, users were more likely to be female, white, married, and enrollees of the commercial insurance Blue Cross Blue Shield. Adoption disparities in gender, race, and socioeconomic status were observed, which is consistent with previous studies on social disparities in enrollment and use of patient portals (see Perzynski et al [27], Graetz et al [28], and Kruse et al [29] and the references therein). Surprisingly, instead of alienating the older generation, young adults aged 19-30 years tended not to adopt patient portals. Members of the younger generation are the habitual users of Web-based applications [30]. It suggests that being accustomed to using Internet and other Web portals may not be a powerful predictor of portal adoption. This is a seemingly counterintuitive observation and might be interpreted as young adults not being strongly motivated to use patient portals because they are healthier and have a relatively low level of health care consumption in general [31]. Being tech-savvy is not the driving force for portal adoption and subsequent usage.

#### Patient Portal Usage

The characteristics of portal adopters were not necessarily the same as those of active portal users. Our findings suggest that the people who potentially enjoy using or need to use patient portals are not aware of, or given enough access to, patient portals. The most important factor driving portal usage intensity is patient disease burden, measured by APN. Patients with a heavy disease burden would use clinical services more frequently and patient portals can provide more convenience for them. Unfortunately, the propensity of portal adoption among the high APN population was not high, although they might be the population that benefits the most from using patient portals. In addition, although Medicare patients did not show a strong intention to adopt portals [27-29,32,33], once they became users, they exhibited a relatively high level of utilization and they were not resistant to using the messaging function of portals for communication. Medicare patients may actually appreciate the value of patient portals but just have barriers to adopt it, which signals a lack of *match* in the patient portal market. External forces, such as incentives, reward programs, and policy initiatives, are needed to channel patients.

Understanding the unique needs and usage habits of different patient populations can contribute to a better and user-friendly design of the portal that can cater its service and functionality to patients’ various tastes and preferences. For instance, an important factor for predicting user types is age. Comparing the younger and the older generations, we found that their attitudes toward using portals to make an appointment or sending a message differed significantly. Older patients did not favor the appointment function as young people did, possibly because most of their appointments are follow-ups and are made directly after their office visits. However, older patients preferred to send messages to their providers compared to other age groups; the relatively high utilization demonstrated the value they found in messaging. This is possibly because they demand frequent and timely communication with their providers, and messaging is a good complement to telephone encounters and office visits to fulfill their heavy needs.

Lastly, the digital divide between races or ethnicities exists not only in adoption, but also in the subsequent use of portals. Nonwhite patients, in general, tended not to adopt nor actively use a portal. In particular, black and African American patients tended not to adopt a portal, and Hispanic patients were very inactive after adopting portals. In particular, Asian patients exhibited a low level of utilization of the messaging function, implying a language barrier [28,34]. It was also found in other research that racial and ethnic minority groups, especially, reported concerns about privacy and information security and they differed from the advantaged (ie, high socioeconomic status) groups in their knowledge and skills of, and comfort in using, the technology, in addition to their accessibility to the technology infrastructure [6,35-37].

Language barriers, poor health literacy, and a low socioeconomic status, among other barriers, contribute substantially to the digital divide. Addressing these barriers will require patient education, infrastructure enhancement, as well as the technological designs that enable patients to communicate with providers in a secure and convenient way [6]. Providers, especially those serving vulnerable populations, should communicate with patients about portal usage and take time to discuss and demonstrate the technology, such as how to use different portal functions. Policy makers and technology developers should ensure the security, privacy, and ease of use of patient portals and the telehealth infrastructure, factoring in the special needs and the concerns of racial and ethnic minority groups. The heterogeneous adoption and usage behaviors of patients signal that the technology acceptance by people is not uniform and can be compounded by multiple factors, such as the conditions to facilitate the use, the ease of use, and the perceived usefulness [38]. Technology adoption theory would play an important role in guiding the design and development of portal functions that benefit patients with different characteristics and care needs.

#### Care Service Utilization and Appointment Adherence

First, the portal usage effects are heterogeneous rather than homogeneous. Different user groups behaved in different ways; ignoring such heterogeneity could lead to misspecification of such effects. Patients who frequently used *messaging and laboratory* together had the largest increase in primary care service utilization, including office visits and telephone encounters, while silent portal users had the largest reduction in using primary care services compared to before adoption. Mixing the two groups will lead to a possible conclusion that the primary care service utilization has not changed, as the positive and negative effects can get cancelled out.

Second, the portal usage effects are dynamic rather than static. Therefore, it is necessary to conduct studies using longitudinal data, not solely relying on observations from cross-sectional studies. The trends of office visit and telephone encounter RRs suggest that the convenience brought by patient portals for supporting better provider-patient interaction might reduce patients’ in-person visits over a longer time frame rather than immediately. This may be due to the fact that patients need time to adapt to portal functionalities, and patient portals influence patients’ health behavior gradually. Both the short-term (ie, a temporary boost) and the long-term (ie, a gradual decline) impacts are critical to informing service operations and guiding policy decisions. Whereas the portal usage was not shown to significantly reduce clinical service consumption immediately, portal activities, such as replying to secure messages, would inevitably increase the provider’s service time. Thus, exploring the payment structure that accommodates the technologically mediated interactions between providers and patients (eg, text messaging, emails, and virtual visits) is instrumental to gaining the buy-in of providers. Policy makers and payers must accordingly recognize and value the amount of time providers spend on interacting and educating patients, particularly the vulnerable and disadvantaged ones, both online and offline.

Lastly, the correct understanding of the heterogeneous and dynamic property of portal usage effects will enable us to carry out targeted and proactive interventions to achieve better patient outcomes. While the user subgroups behaved differently toward their health care consumption, the no-show rates of portal users were, overall, lower compared to that of nonusers, with different magnitudes of change. It also reveals that actively using patient portals, in contrast to being silent, leads to a larger improvement in appointment adherence. To achieve better patient engagement, providers can take the initiative in messaging patients, especially the ones with a high APN, to stimulate their portal usage and, thus, to raise their awareness of care engagement. In addition, elements of gamification can be imbedded into portal functions to encourage and reward patients. Virtual rewards or incentives can be made to patients who exhibit a high level of portal interactions (eg, actively reading the after-visit summary, physicians’ notes, and lab results, as well as participating in portal-based surveys, such as quality-of-care questionnaires and patient-reported outcomes).

### Limitations

There are several limitations to our study. First, our causal inference analysis was based on an observational study. Admittedly, no unmeasured confounder is typically assumed to identify causal effects and is difficult to validate. However, even with unmeasured confounders, as long as they are time-invariant, their effects will be “cancelled” owing to the DID study design with the before-after comparison. If confounders are time-varying and measurable, we can treat them in the same fashion as dealing with a patient’s APN (ie, a time-varying confounder) and their disease process (ie, conditioning on their APN and whether a new disease occurred at each quarter). To test the robustness of our framework against unmeasured time-varying confounders, in future work, we plan to develop a trend-in-trends study [39]. In addition, we propose to design a synthetic control test [40] as a sensitivity analysis to evaluate the impact of any unmeasured confounders to our major results. Second, the dataset was limited to UF Health patients. We presented the demographics and socioeconomic characteristics of our patient population as a one-site study so that the results can be compared to other systems with the same or a different patient makeup. We also examined the institutional evaluation and management codes, which represent the severity level of patients: the larger the number, the more complicated the service [41]. It confirmed that our patient population shared a similar *patient level* in terms of severity to other academic health systems. Therefore, we hope that this patient population can be considered as being representative to generate a general insight. Third, the causal effects of portal usage on patient-reported outcomes and other adherence behaviors (eg, medication adherence) cannot be established using the data collected for this study. Also, we were not able to assess the impact of portal usage on patients’ specialty care consumption and their out-of-the-network urgent care and emergency department visits. We plan to expand the data spectrum to include some of these outcomes as part of the future work. Lastly, the business value and economic impact of patient portal implementation need to be quantified.

### Conclusions

In closing, patients differ in their portal adoption and usage behaviors, and the portal effects are heterogeneous and dynamic. There exists a lack of *match* in the patient portal market in the sense that patients who benefit the most from using patient portals are not actively adopting patient portals. Patient portal usage was confirmed as effective in reducing appointment no-shows. However, to maximize the potential of patient portals, it is paramount to understanding the value that patient portals could bring to patients who have exhibited different characteristics and care needs. Health care delivery planners and administrators should, on the one hand, remove the barriers of adoption for the portal beneficiaries and, on the other hand, incorporate the impact of portal usage into care coordination and workflow design, ultimately aligning patients’ and providers’ needs and functionalities to enhance care delivery.

## Appendix

Multimedia Appendix 1 Illustration of two-stage clustering and descriptive statistics of the clustering results.

## Abbreviations

APN: active problem number
APPT: appointment
DID: difference-in-differences
EMR: electronic medical record
HINTS: Health Information National Trends Survey
LAB: laboratory
MED: medication
MESG: messaging
MLR: multinomial logit regression
N/A: not applicable
OR: odds ratio
Q1: first quarter, January-March
Q2: second quarter, April-June
Q3: third quarter, July-September
Q4: fourth quarter, October-December
RR: rate ratio
UF: University of Florida
