# Supplementary material for: Characteristics of Patients Using Different Patient Portal Functions and the Impact on Primary Care Service Utilization and Appointment Adherence: Retrospective Observational Study
Source: J Med Internet Res. 2020 Feb 25;22(2):e14410. doi: 10.2196/14410 (PMC7064955; doi:10.2196/14410)
Supplement: Multimedia Appendix 1 [file jmir_v22i2e14410_app1.docx]

## Appendix

### Two-Stage User Subgroup Clustering

Figure A1. An illustration of the two-stage user subgroup clustering. (a) First stage. (b) Second stage.


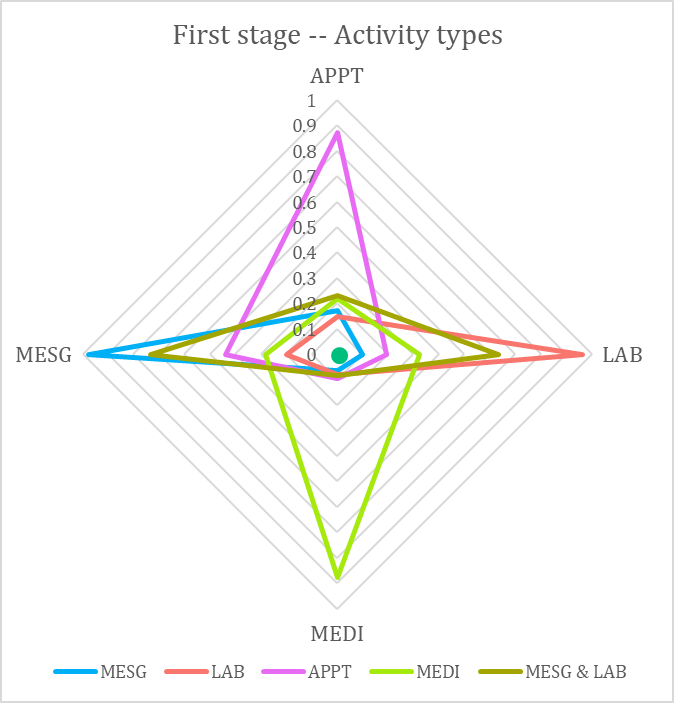

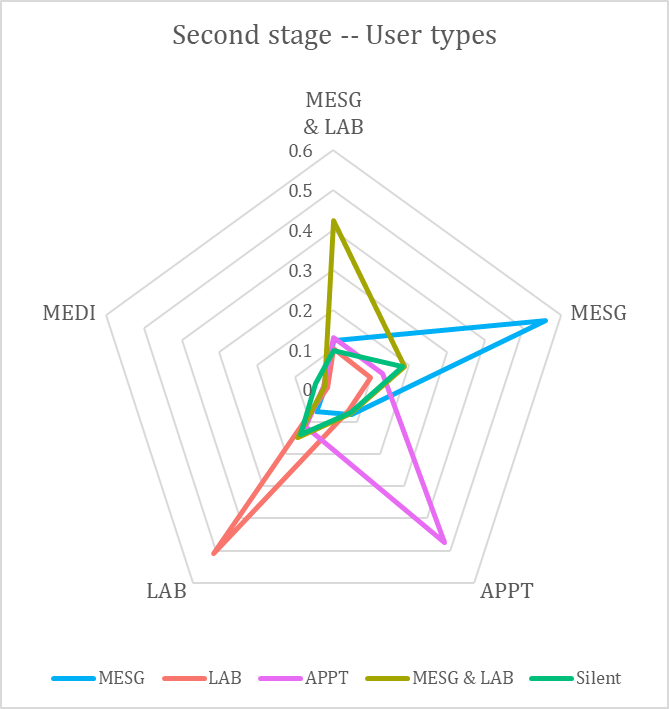


The figure on the left hand side presents how the relative use (normalized according to the spherical clustering method with cosine similarity) of the four major portal activities is distributed for each activity cluster. Each quadrilateral represents one cluster. We obtained five activity clusters, $C_{MESG}$, $C_{LAB}$, $C_{MED}$, $C_{APPT}$, and $C_{M\&L}$. In addition, there is an inactive cluster $C_{Silent}$ in the middle. The figure on the right hand side shows how the relative occurrences of the different activity clusters are distributed for each user type. Five user types, $U_{MESG}$, $U_{LAB}$, $U_{APPT}$, $U_{M\&L}$, and $U_{Silent}$.were defined in the second stage. All clusters are color-coded and described in their corresponding legend.

Table A1. Distributions of portal activities for each user subgroup.

| Mean (per quarter)  (95% CI) | | Portal Usage Types | | | | |
| --- | --- | --- | --- | --- | --- | --- |
|  |  | $U_{MESG}$ | $U_{LAB}$ | $U_{APPT}$ | $U_{M\&L}$ | $U_{Silent}$. |
| Activities | APPT | 7.48 | 6.07 | 21.35 | 10.80 | 3.10 |
|  |  | (7.12, 7.84) | (5.53, 6.60) | (20.13, 22.56) | (10.21, 11.40) | (2.92, 3.28) |
|  | LAB | 8.81 | 22.57 | 14.23 | 22.51 | 6.32 |
|  |  | (8.38, 9.24) | (21.23, 23.91) | (13.24, 15.22) | (21.5, 23.53) | (6.03, 6.62) |
|  | MEDI | 1.77 | 1.73 | 2.92 | 2.38 | 1.06 |
|  |  | (1.69, 1.85) | (1.62, 1.84) | (2.74, 3.10) | (2.26, 2.51) | (1.00, 1.12) |
|  | MESG | 29.20 | 11.01 | 19.05 | 27.05 | 8.22 |
|  |  | (28.21, 30.2) | (10.19, 11.83) | (18.04, 20.07) | (25.94, 28.15) | (7.85, 8.58) |

The table articulates that, after the patient-level clustering, the distributions of the overall portal activities among user subgroups are indeed different, and they match their corresponding labels. For instance, for the user subgroup $U_{MESG}$, patients therein had a significant larger amount of message usage compared to the other portal functions such as appointment, lab, and medication (vertical comparison), and the usage of messaging was also significantly larger than that of patients in other user subgroups, such as $U_{LAB}$, $U_{APPT}$, and $U_{Silent}$ (horizontal comparison).

### Panel Difference-in-Differences (DID) Model

Let index $t$ denote the time the measurements (responses and covariates) were recorded, $t=0,1,\ldots,T$, where $t=0$ represents the beginning quarter of the study period, and $T=11$. $Time_{i\tau(t)}$ is a dummy variable to adjust for time or seasonal effect $\lambda_{\tau}$, and $Time_{i\tau(t)}=1$ if and only if $\tau=t$, $t=0,\ldots,T$. To address the confounding effects, discretized APN is incorporated as a time-varying covariate ${APN}_{it}$ for the $i^{th}$ patient at time (quarter) $t$. Patients could have their new disease onset visits during any $t$ such that $0\leq t\leq T$. To incorporate the disease process that affects both users and nonusers, a dummy variable $D_{ik(t)}$ is introduced to represent if the absolute time $t$ is $k$ quarters post patient $i's$ new diagnosis. The disease effect at time lag-k, $k=0,\ldots,T$ is captured by coefficient $\theta_{k}.$ To investigate the time trend in heterogeneous treatment (user subgroup) effects, a dummy variable $P_{iuk(t)}$ is introduced, and $P_{iuk(t)}=1$ if and only if the absolute time $t$ is $k$ quarters post patient $i's$ portal adoption, and the patient belongs to user subgroup $u$. Consequently, $\beta_{ku}$ is the lag-k treatment effect of treatment type $u$, i.e., the difference-in-differences between the user subgroup $u$ and nonusers at the $k^{th}$ quarter post portal adoption. Other time-invariant covariates (age, gender, race, marital status, and insurance type) are represented by $X_{i}$, for the $i^{th}$ patient. To accommodate the special variance structure of count responses, a generalized linear model with *Poisson distribution adjusted for over-dispersion* is proposed:

$${g(E[Y}_{it}])={\gamma User}_{iu}+\sum_{\tau=0}^{T} \lambda_{\tau}Time_{i\tau(t)}+\sum_{k=0}^{T} \theta_{k}D_{ik\left( t \right)}+\sum_{u=0}^{U} \sum_{k=0}^{T-1} \beta_{ku}P_{iuk\left( t \right)}+\alpha{APN}_{it}+\eta X_{i}. (1)$$

In model (1), $g(\cdot)$ is a log-link function, $Y_{it}$ is the outcome variable (i.e., number of office visits arrived, cancelled, and no-show, as well as number of telephone encounters for the $i^{th}$ patient at time $t$), and $E[Y_{it}]$ represents the expectation of the corresponding outcome. ${User}_{iu}$ is a dummy variable to indicate whether the $i^{th}$ patient belongs to the user subgroup $u$ or not. If $Y_{it}$ represents the number of office visits, then, the office visit rate ratio (RR) of the users to the matched nonusers is calculated as$\exp^{\beta_{ku}}$. If $\exp^{\beta_{ku}}<1$, then, it can be concluded that using portals is associated with a reduced office visit rate.

### No-Show and Cancellation Comparisons

No-show rate is defined as the number of appointment no-shows divided by the number of appointments scheduled for a given duration. At patient-level, we denote the number of appointment no-shows for patient $i$ at the $k^{th}$ quarter post adoption as $Y_{ik(t)}$, and denote the number of appointments made that were not no-show as $Z_{ik(t)}$. $P_{iuk(t)}$ denotes whether time $t$ is the $k^{th}$ quarter post adoption of user type $u$, and $X_{i}$ and ${APN}_{it}$ are defined the same as above. For type $u$ users at the $k^{th}$ quarter post intervention, we are interested in the patient-level no-show rate denoted as

$$r_{iuk(t)}=\frac{{E[Y}_{ik(t)}\left| P_{iuk\left( t \right)}=1,{{APN}_{it},X}_{i} \right]}{{E[Y}_{ik(t)}\left| P_{iuk\left( t \right)}=1,{{APN}_{it},X}_{i} \right]+{E[Z}_{ik(t)}\left| P_{iuk\left( t \right)}=1,{{APN}_{it},X}_{i} \right]}. (2)$$

For brevity purposes, we omit the quarter index $k$ and time index $t,$ and introduce $r_{i}^{1}$ and $r_{i}^{0}$ to represent a specific user type’s no-show rate and a counterfactual case (if that patient is a nonuser):

$$r_{i}^{1}=\frac{{E[Y}_{i}\left| P_{i}=1,X_{i} \right]}{{E[Y}_{i}\left| P_{i}=1,X_{i} \right]+{E[Z}_{i}\left| P_{i}=1,X_{i} \right]}, (3)$$

$$r_{i}^{0}=\frac{{E[Y}_{i}\left| P_{i}=0,X_{i} \right]}{{E[Y}_{i}\left| P_{i}=0,X_{i} \right]+{E[Z}_{i}\left| P_{i}=0,X_{i} \right]}. (4)$$

Then, the no-show rate ratio of patient $i$ comparing before and after portal adoption is denoted as $RR_{i}=\frac{r_{i}^{1}}{r_{i}^{0}}.$ Next, we introduce another set of variables: $\rho_{i}^{1}=\frac{{E[Y}_{i}\left| P_{i}=1,X_{i} \right]}{{E[Z}_{i}\left| P_{i}=1,X_{i} \right]},$ $\rho_{i}^{0}=\frac{{E[Y}_{i}\left| P_{i}=0,X_{i} \right]}{{E[Z}_{i}\left| P_{i}=0,X_{i} \right]},$ and $\Omega_{i}=\frac{\rho_{i}^{1}}{\rho_{i}^{0}}.$

Variables $\rho_{i}^{1}$ and $\rho_{i}^{0}$ can be roughly interpreted as the average number of no-shows that will occur to successfully complete one appointment. It can be easily proved that $RR_{i}<1$iff. $\Omega_{i}<1$. If the no-show rate is reduced after portal adoption, then $\Omega_{i}<1$, and vice versa. Therefore, testing the hypothesis H_0_: $RR_{i}<1$ is equivalent to test the hypothesis H_0_^’^: $\Omega_{i}<1$. Specifically, let $\beta_{ku}$ be the difference-in-differences of appointment no-shows and $b_{ku}$ be difference-in-differences of appointments made that were not no-show between the type $u$ users and nonusers at the $k^{th}$ quarter post adoption. If $\exp^{\beta_{ku}-b_{ku}}<1$, then, it can be concluded that using portals is associated with a reduced no-show rate. The differences of coefficients of treatment effects between the two models, i.e., $\beta_{ku}-b_{ku}$,$k=0,\ldots,T-1$, can be compared and formally tested. Similarly, $Y_{ik(t)}$ can be the number of appointment cancellations for patient $i$ at quarter $k$ post intervention to investigate the ratio of cancellation rate in the user subgroups to that in the nonuser group.
